# Microbial Biofilms and Antibiofilm Agents 2.0

**DOI:** 10.3390/ijms23147932

**Published:** 2022-07-19

**Authors:** Giovanna Batoni, Giuseppantonio Maisetta, Semih Esin

**Affiliations:** Department of Translational Research and New Technologies in Medicine and Surgery, University of Pisa, 56123 Pisa, Italy; giuseppantonio.maisetta@unipi.it (G.M.); semih.esin@unipi.it (S.E.)

It is estimated that <0.1% of the total microbial biomass on the Earth is present as individual, free floating (i.e., planktonic) cells. Nevertheless, it is only in 1993 that the American Society for Microbiology recognized the relevance of biofilms (i.e., aggregates of microbial cells attached to a substrate and embedded in a self-produced extracellular matrix) in the medical field [1]. Since then, thousands of studies have been conducted in order to understand how microbial biofilms develop and regulate as well as how we can limit their growth or eradicate them. Despite this intensive work, the therapy of biofilm-associated infections is still problematic to date and relies on the use of anti-adhesive or antibacterial coatings, on early aggressive antibiotic-treatments to prevent biofilm formation, or on the administration of high doses of systemic antibiotic combinations to eradicate pre-formed biofilms (Figure 1). If all these measures fail, the only option is the surgical removal of the infected tissue or of the implant with suffering for the patients and high healthcare costs. The great knowledge on biofilms that is being accumulated represents, however, a solid background to look to the future with optimism regarding our ability to implement innovative antibiofilm strategies to control harmful biofilms (Figure 1). In this context, the aim of this Special Issue was to gather a collection of papers focused on biofilm infections, and on the development of new antibiofilm agents to target them. A total of eight research articles and two reviews were collected disclosing new and interesting aspects on the physiology of bacterial biofilms [2], biofilm interaction with host cells [3], and innovative and promising approaches to prevent or eradicate microbial biofilms, not only in the medical setting [4,5,6,7,8,9,10] but also in the environment [11].

The contribution of Rahman et al. [2] focuses on how the proteome of *Staphylococcus aureus* biofilms changes with aging compared with planktonic growth. The theme is very relevant as in vitro studies and clinical interventions suggest that biofilm’s treatment failure increases with biofilm maturity/thickness [12,13]. Therefore, the identification of age-dependent biofilm signatures may help to disclose targets to interfere with the development of early as well as aged biofilms.

The complex interplay between *S. aureus* biofilms and host cells in a wound is the topic addressed by Czajkowska et al., in their work [3]. The Authors established an interesting in vitro human fibroblast: *S. aureus* biofilm coinfection model resembling the phase of critical colonization of an infected wound (i.e., the tipping point that requires medical intervention to stop the progression to severe infection). At such a crucial phase, they analyzed via 1H-NMR spectroscopy the metabolites (of both bacterial and host-cell origin) released in the co-culture supernatant, considered by the Authors to be a surrogate of the wound exudate. Such analysis allowed the identification of two extracellular metabolites (lactate and acetate) that were characteristic only for staphylococcal biofilm co-cultured with fibroblast cells, but not for fibroblast cells alone. The study and the results obtained might have important clinical implications as they may lead to innovative diagnostic procedures for the rapid and indirect detection of *S. aureus* biofilm in a wound, increasing the efficacy of its removal with the use of antiseptics.

As pointed out by Martin et al. in their review focused on the importance of biofilms in the context of cystic fibrosis, two different biofilm components may be targeted by an antibiofilm agent: the bacterial cells themselves, and the complex matrix that embeds and protects biofilm cells, commonly referred to as extracellular polymeric substance (EPS) [4]. This latter consists of polymers of different nature, including polysaccharides, proteins, extracellular DNA as well as ions and small molecules that play a major role in maintaining biofilm architecture, ensuring a highly hydrated environment and, most of all, in reducing antibiotic effectiveness against biofilms [14]. Thus, agents that digest or inhibit this protective layer may help to dislodge bacterial cells, better exposing them to antibiofilm agents and host immune system. The review highlights that, in addition to these targets, the complex signaling pathways that govern biofilm development might also offer interesting possibilities of therapeutic interventions against biofilms [4].

Recent literature points to metal complexes as promising leads for the development of effective antibiofilm agents due to their multi-target and complex mechanisms of actions [15]. Two articles of the collection deal with this topic: in the first one, the Authors evaluated the antibacterial and antibiofilm activity of three ruthenium (Ru) complexes in different oxidation states, with 2-Pyridin-2-yl-1 H-benzimidazole [5], in the second manganese(II) complexes with heteroaromatic ligands (i.e., alcohol, aldehyde, ketone, and carboxylic acid) were investigated [6]. Interestingly, the antibiofilm activity of Ru complexes was found to increase as compared to that of the ligand alone and was demonstrated to be possibly due to an effect on the hydrophobicity of the bacterial surface that, in turn, reduced the adhesive properties of the treated bacteria (*Pseudomonas aeruginosa* PAO1) [5]. In the case of manganese complexes, again, an increased activity against *P. aeruginosa* PAO1 biofilms was observed as compared to the free ligands or manganese(II) salts, together with morphological changes in the biofilm structure and reduction in the secretion of pyoverdine, one of the major virulence factor of *P. aeruginosa* [6]. In both studies, a good biocompatibility of the active complexes was demonstrated.

Pusparajahand and coworkers reviewed the emerging strategies against biofilms of methicillin-resistant *S. aureus* (MRSA), one of the main and difficult to treat bacterial species involved in biomedical device-related infections [7]. In particular, the review focused on the multitude and still under-investigated anti-staphylococcal/MRSA biofilm metabolites that may derive from *Streptomyces* spp. bacteria isolated from different environmental sources, and on their mechanisms of biofilm inhibition and/or eradication.

Although much less investigated than bacterial biofilms, also fungal biofilms play a major role in human infections [16]. One of the articles from the collection [8] evaluated the effect of three imidazolium-based ionic liquids, incorporated with the natural terpene alcohol 1R,2S,5R)- (−)-menthol, on fungi of the genera *Candida*, commonly found in long-time denture wearers. In the presence of predisposing factors (e.g., immunosuppression, poor oral hygiene) such fungi and in particular *C. albicans* might be involved in the onset of *Candida*-associated denture stomatitis due to their ability to adhere to the acrylic resins of the dental prostheses and form biofilms [17]. Interestingly, all the compounds tested in the study inhibited *C. albicans*-biofilm formation on acrylic, and partially on porcelain and alloy dentures. The possible mechanisms of biofilm eradication were found to be dependent on hyphae inhibition (for alkyl derivatives) or cell wall lysis and reduction in the adhesin level (for alkyloxymethyl derivative). Due to the demonstrated activity, and low toxicity towards mammalian cells, the Authors propose the tested compounds as potential antifungal denture cleaners.

Development of appropriate drug delivery systems for conventional (e.g., antibiotics, antiseptic molecules) and unconventional antibiofilm agents is another direction to follow assiduously to make progress in our ability to control biofilms. Two articles of the collection deal with the use of bacterial cellulose (BC) as a delivery system for antibiofilm active molecules [9,10]. Such biopolymers are produced by numerous genera of bacteria and are emerging as promising candidates for the delivery of active pharmaceutical compounds due to their favorable physical, chemical and biological properties that include flexibility, high porosity, hydrophilic nature, small size, high polymerization degree, easy modification, biodegradability, and biocompatibility [18]. In the first article of the collection, the Authors chemisorbed BC with several antiseptic molecules, commonly used for the management of biofilm-associated wound infections, and evaluated the anti-biofilm activity of such dressings against a vast collection of strains involved in wound infections [9]. High levels of antibiofilm activity were recorded, especially when BC was functionalized with iodine povidone whose action was higher than that of a conventional broadly applied silver dressing, at least against some of the bacterial species tested. In the second article, from the same group [10], BC coupled with gentamicin is proposed as an absorbent layer of surgical meshes to prevent infection emergence after hernia repairs. The BC-modified meshes were easily colonized by fibroblasts (in a faster manner and at higher levels than native meshes) and released higher concentrations of gentamicin as compared to uncoated meshes, allowing in vitro eradication of *S. aureus*.

In the era of ecological transition and environmental conservation, the identification of sustainable (green) antibiofilm strategies is becoming increasingly important and a key field of biofilm research. In this context, the Special Issue includes an article dealing with the control of environmental biofilms [11]. In the article, an interesting solution to the problem of marine biofouling, referred to as the attachment and proliferation of microorganisms on immersed surfaces, is addressed. In particular, a natural and eco-friendly three-dimensional carbon skeleton material, treated rape pollen (TRP), is proposed in the article for biofouling control, owing to its visible-light-driven photocatalytic disinfection property.

In conclusion, significant advances have been made in recent decades in our understanding of microbial biofilms and in the development of measures to counteract their development. The experience accumulated is suggesting that effective solutions to the complexity of the biofilm world are on the horizon, and do not involve the massive use of conventional antibiotics as also corroborated in this Special Issue in which most of the proposed strategies are not antibiotic based. It is also becoming evident that the problem of biofilms can only be tackled by using interdisciplinary approaches that involve different expertise from clinicians, microbiologists, biomedical engineers, bio-materialists, chemists and molecular biologists. The combination of various skills will allow a holistic approach to the problem and, hopefully, will help to fill the gaps that still separate us from an efficient biofilm control.

## Figures and Tables

**Figure 1:**
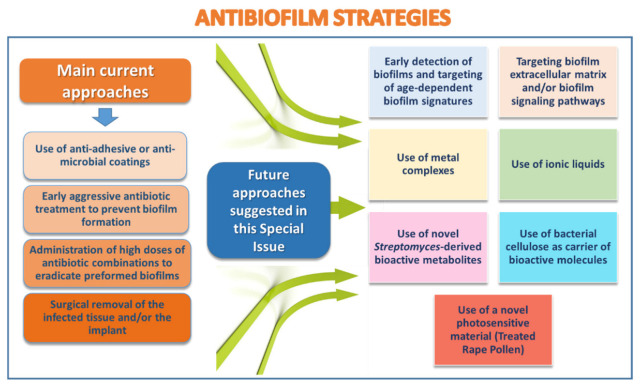
Current and developing anti-biofilm approaches.
